# The 2015 Bioinformatics Open Source Conference (BOSC 2015)

**DOI:** 10.1371/journal.pcbi.1004691

**Published:** 2016-02-25

**Authors:** Nomi L. Harris, Peter J. A. Cock, Hilmar Lapp, Brad Chapman, Rob Davey, Christopher Fields, Karsten Hokamp, Monica Munoz-Torres

**Affiliations:** 1 Lawrence Berkeley National Laboratory, Berkeley, California, United States of America; 2 The James Hutton Institute, Dundee, United Kingdom; 3 Center for Genomic and Computational Biology, Duke University, Durham, North Carolina, United States of America; 4 Bioinformatics Core, Harvard School of Public Health, Boston, Massachusetts, United States of America; 5 The Genome Analysis Centre, Norwich, United Kingdom; 6 National Center for Supercomputing Applications, University of Illinois Urbana-Champaign, Urbana, Illinois, United States of America; 7 Smurfit Institute of Genetics, Trinity College Dublin, Dublin, Ireland

## Abstract

The Bioinformatics Open Source Conference (BOSC) is organized by the Open Bioinformatics Foundation (OBF), a nonprofit group dedicated to promoting the practice and philosophy of open source software development and open science within the biological research community. Since its inception in 2000, BOSC has provided bioinformatics developers with a forum for communicating the results of their latest efforts to the wider research community. BOSC offers a focused environment for developers and users to interact and share ideas about standards; software development practices; practical techniques for solving bioinformatics problems; and approaches that promote open science and sharing of data, results, and software. BOSC is run as a two-day special interest group (SIG) before the annual Intelligent Systems in Molecular Biology (ISMB) conference. BOSC 2015 took place in Dublin, Ireland, and was attended by over 125 people, about half of whom were first-time attendees. Session topics included “Data Science;” “Standards and Interoperability;” “Open Science and Reproducibility;” “Translational Bioinformatics;” “Visualization;” and “Bioinformatics Open Source Project Updates”. In addition to two keynote talks and dozens of shorter talks chosen from submitted abstracts, BOSC 2015 included a panel, titled “Open Source, Open Door: Increasing Diversity in the Bioinformatics Open Source Community,” that provided an opportunity for open discussion about ways to increase the diversity of participants in BOSC in particular, and in open source bioinformatics in general. The complete program of BOSC 2015 is available online at http://www.open-bio.org/wiki/BOSC_2015_Schedule.

**Figure:**
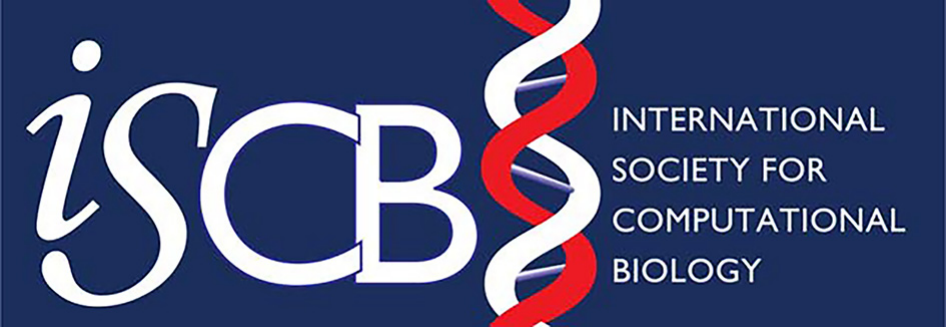


## Introduction

The 16th annual Bioinformatics Open Source Conference (BOSC 2015, http://www.open-bio.org/wiki/BOSC_2015) was held in Dublin, Ireland, in July 2015. Co-chaired by Nomi Harris and Peter Cock, the conference brought together over 125 bioinformatics researchers, developers, and users of open source software. BOSC is organized by the Open Bioinformatics Foundation (OBF), a nonprofit group dedicated to promoting the practice and philosophy of open source software development and open science within the biological research community. The OBF was one of the founding International Society for Computational Biology (ISCB) Communities of Special Interest (COSI), a program formally launched in 2014. Since its beginning in 2000, BOSC has been run as a two-day special interest group (SIG) before the annual ISMB conference.

BOSC is a forum for bioinformatics developers to communicate the results of their latest efforts to the wider research community [1]. The conference also provides a focused environment in which developers and users can interact and share ideas about standards, software development practices, and practical techniques for solving bioinformatics problems. The scope of BOSC encompasses the wide range of open source bioinformatics software being developed and also includes the growing movement of open science, which emphasizes transparency, reproducibility, and data provenance. Topics covered typically include new computational methods, reusable software components, visualization, interoperability, and other approaches that help to advance research in the biomolecular sciences.

Open source software has flourished in the bioinformatics community since the 1990s. When BOSC first began, the concept of open source software was new and still controversial in computational science. One of BOSC’s initial objectives was therefore to showcase and promote the value of the open source model of software development for bioinformatics. This has arguably been thoroughly accomplished—open source licensing has become common for bioinformatics software, and the merits of open source as a model are rarely debated anymore. As a result, BOSC widened its scope to encompass open science as a whole, of which open source is one aspect.

BOSC includes two days of talks, posters, a panel discussion, and “Birds of a Feather” (BOF) interest groups. Session topics this year included “Data Science;” “Standards and Interoperability;” “Open Science and Reproducibility;” “Translational Bioinformatics;” “Visualization;” the traditional session on “Bioinformatics Open Source Project Updates;” and a session for late-breaking five minute lightning talks. In addition to two keynote talks, the program included 19 normal-length (15 minutes) talks and 24 lightning talks, as well as 33 posters. The complete program is available online at http://www.open-bio.org/wiki/BOSC_2015_Schedule. Links to articles, blog posts, and Twitter summaries (there were over 2,000 Tweets about #BOSC2015) can be found there as well. Most of the slides and posters from BOSC 2015 are hosted on an F1000 Research channel (http://f1000research.com/channels/BOSC), and talk videos can be found on the BOSC YouTube playlist (https://www.youtube.com/playlist?list=PLir-OOQiOhXbENjAIFF-JZ0WodnysPqfh).

## Panel

In recent years, BOSC has included a panel discussion that offers all attendees the chance to engage in conversation with the panelists and each other. In 2015, the panel was titled “Open Source, Open Door: Increasing Diversity in the Bioinformatics Open Source Community,” and focused on the important topic of what can be done to increase the diversity of participants in BOSC in particular, and in open source bioinformatics in general. Chaired by Monica Munoz-Torres, the panel included Holly Bik (who was also one of the keynote speakers), Michael R. Crusoe, Aleksandra Pawlik, and Jason Williams (see Fig 1). The panel arose as a follow-up to a 2014 BOF session on the diversity issue and aimed to solicit actionable ideas about what we can do to make everyone feel welcome, both at BOSC and in the communities of its constituent open source projects.

**Fig 2:**
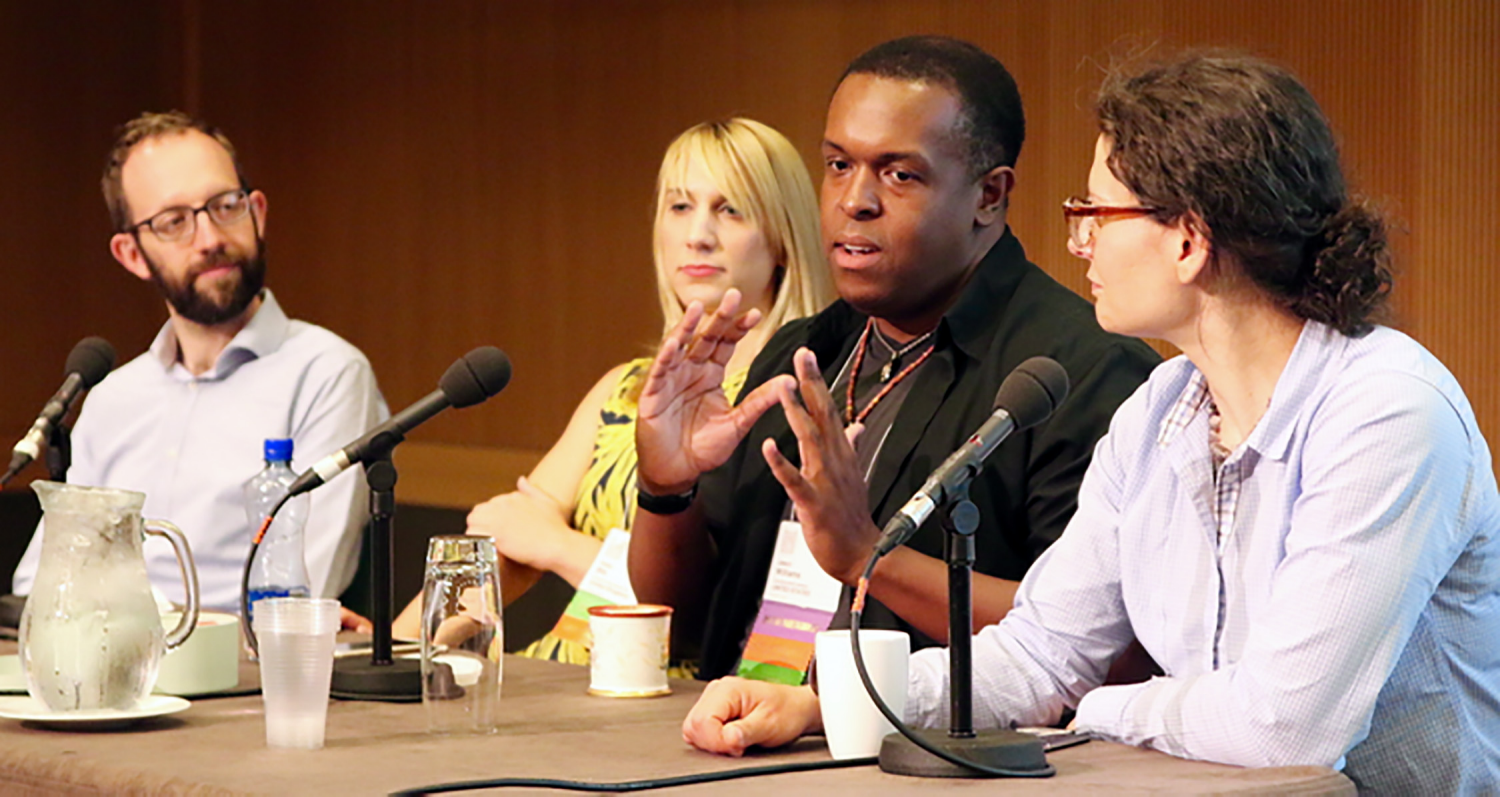
2015 BOSC panelists (from left): Michael R. Crusoe, Holly Bik, Jason Williams, and Aleksandra Pawlik.

## Keynote Talks

Keynote talks by researchers who are influential in some aspect of open source bioinformatics are a popular part of BOSC. Past speakers include bioinformatics luminaries such as Phillip Bourne, Sean Eddy, Jonathan Eisen, and Carole Goble. keynote speakers at BOSC 2015 were Holly Bik and Ewan Birney.

Holly Bik is a Birmingham Fellow (Assistant Professor) in the School of Biosciences at the University of Birmingham, United Kingdom. Her research uses high-throughput environmental sequencing approaches (rRNA surveys, metagenomics) to explore biodiversity and biogeographic patterns in microbial eukaryote assemblages. In her keynote talk, titled “Bioinformatics: Still a Scary World for Biologists,” Dr. Bik discussed her transition from biology to bioinformatics and what some of the barriers were. This provided a valuable perspective to conference attendees who are largely focused on bioinformatics and may be less aware of how to make their software tools accessible to biologists who aren’t programmers.

Ewan Birney is Joint Director of the European Bioinformatics Institute, and is known for his role in annotating the human, mouse, and other genome sequences. He led the analysis group for the Encyclopedia of DNA Elements (ENCODE) project, which aims to define functional elements in the human genome. He was also one of the early leaders of the BioPerl project, and a cofounder of the OBF. In his talk, “Big Data in Biology,” Dr. Birney began by providing a high-level picture of the importance of sequencing technology and the impact its evolution has had on biology and bioinformatics. He went on to discuss why open source matters; how deeply we rely on scalable infrastructure; and how information might be stored efficiently in DNA.

## Birds of a Feather Sessions

To foster communication and collaboration between attendees, BOSC encourages attendees to organize “Birds of a Feather” sessions, which are focused but informal discussions about a topic of shared interest. Although the BOFs themselves are only about an hour long, they often catalyze conversations and projects that continue beyond BOSC. For example, the 2014 BOF about increasing diversity inspired BOSC’s organizers to implement some of the ideas that arose during the discussion, including appointing an outreach coordinator. In 2015, one BOF concentrated on the Common Workflow Language (CWL), an industry/academia collaboration that came together during the 2014 pre-BOSC Codefest (see the section on “Affiliated Events”).

Another BOF session at BOSC 2015 met on both days, in the form of an “unconference” [2], to identify and discuss approaches for building successful open source bioinformatics developer communities (http://www.hub-hub.de/wiki/index.php?title=BOSC2015Unconf). The organizers of the BOF have since set up a GitHub repository to continue soliciting community input, and to collaboratively author an article that presents the findings.

## Encouraging Diversity at BOSC

Inclusivity was one of the founding principles of BOSC, and a nondiscrimination clause has been included from the outset in the bylaws of OBF, the organization that runs the conference. In an effort to more effectively promote diversity in BOSC and the bioinformatics open source community at large, we have recently stepped up our inclusivity endeavors (see [3] for a more in-depth report). Diversity in this context encompasses many aspects, including academic background, biological specialty, gender, ethnicity, age, and geographical location. We successfully lobbied the ISCB to adopt a code of conduct across its branded conferences; reached out to organizations that support groups that are underrepresented in bioinformatics and open source software to promote BOSC to a broader audience; and sponsored registration for some speakers who would otherwise have faced financial barriers to attend BOSC. Based on a survey of the room, around half of the BOSC 2015 attendees were there for the first time, evidence that the outreach efforts have made a positive impact.

In addition to the diversity panel described above, the 2015 meeting included smaller changes aimed at boosting participation by all attendees, such as giving attendees the option to ask questions after talks, anonymously, by writing them on notecards (an idea proposed by panelist Michael R. Crusoe, who was inspired by a blog post by Valerie Aurora [4]) or tweeting them to us. This approach seemed to be successful at encouraging questions from people who might not have felt comfortable standing up and asking them, and we plan to continue it at future BOSCs.

## Affiliated Events

Since 2010, we have organized a two-day collaborative community development event prior to BOSC, called Codefest [5]. The event is open to anyone interested, has no registration costs, and provides a venue for open source bioinformatics developers to meet in person to work on or plan joint projects. Codefest 2015 (http://www.open-bio.org/wiki/Codefest_2015) was held at Trinity College, Dublin, and was attended by over 30 people who worked together on projects that included extensions to the CWL (which was initiated at the 2014 Codefest), increased support of Galaxy [6] and CWL for EDAM [7], and improvements to parsing and debugging in Biopython [8].

For the years when ISMB and BOSC are held in North America—which makes attending Codefest more difficult for developers based in Europe—EU-Codefests, similar in format and spirit to the BOSC-affiliated Codefests, have been held. Occasionally, BOSC also partners with other organizations to co-organize collaborative development-oriented events, such as the BOSC/Broad Interoperability Hackathon that was held in April 2013.

## BOSC 2016

BOSC 2016 will take place in Orlando, Florida, United States, on July 8–9, just before the ISMB 2016 meeting. BOSC is run by volunteers; those interested in helping out are encouraged to contact the organizing committee at bosc@open-bio.org.
